# Stammering

**Published:** 1887-08

**Authors:** 


**Stammering.**—Dr. G. H. Treadgold, in *Medical World:* "If Dr. O. Baum will instruct his patients to hold a piece of cork or any hard substance between the molar teeth while talking, so as to keep the lower jaw immovable, and tone up the nervous system, his patient will soon be rid of their trouble. Belladonna has some remedial properties in small doses."

